# Novel 3D printed universal conical holder for eye plaque quality assurance

**DOI:** 10.1002/acm2.14395

**Published:** 2024-05-14

**Authors:** Wentao Wang, Jacqueline Emrich, Firas Mourtada

**Affiliations:** ^1^ Thomas Jefferson University Philadelphia Pennsylvania USA

**Keywords:** 3D printing, eye plaque, quality assurance

## Abstract

**Purpose:**

For the custom‐built construction of eye plaques, the iodine (I‐125) seeds of different source strengths are recycled in our eye plaque program. To return I‐125 seeds to the correct lot, we developed a novel 3D‐printed conical plaque QA holder for relative assay for eye plaques.

**Materials and methods:**

A universal 3D‐printed conical plaque holder was designed to accommodate six plaque sizes and fit reproducibly in a well‐type dose calibrator. A reproducibility test was used to compare the plaque placement consistency in the holder versus without the holder. Plaque assays were performed for assembled plaques both before implant and after explant. The explant reading was compared with the implant reading adjusted for decay, and the relative error was calculated. The plaque response fraction (PRF) is defined as the fraction of well chamber implant reading over the total seed strength for a plaque. The PRF was aggregated for each individual plaque to confirm the seed lot before implant.

**Results:**

The reproducibility test showed the chamber reading's relative standard deviation of 0.40% with the QA holder compared to 0.68% without it. The batch relative assay was performed for 251 plaques. The absolute value of measurement deviation between explant and decay‐corrected implant readings is 0.89% ± 0.86% (mean ± standard deviation). The PRFs for individual plaques range from 36.49% to 49.87%, with a maximum standard deviation of 2%.

**Conclusions:**

This novel 3D‐printed QA holder provides reproducible setup for assaying assembled eye plaques in a well chamber. Batch relative assay can validate the seed batch used and plaque integrity during the implant without assaying individual seeds, saving valuable physicist time and radiation exposure from seed handling.

## 1 INTRODUCTION

Eye plaque brachytherapy commonly utilizes low‐energy radioactive seeds, such as iodine (I‐125) (half‐life: 59.4 days), assembled on a gold alloy plaque to treat ocular melanoma. The Thomas Jefferson University/Wills Eye Hospital plaque program has a typical weekly patient load of 5−7 plaque insertions. For the required custom‐built construction of these plaques on a weekly basis, the I‐125 seeds of different source strengths are recycled after each use. According to our previous analysis,^1^ each seed was used 2.14 times on average prior to being returned to vendor, which resulted in cost savings of $576 per plaque compared to ordering new seeds for each plaque.

AAPM TG‐221^2^ recommends that > 10% or 10 loose non‐sterile sources should be assayed by the institution preceding clinical use. While the U.S. Nuclear Regulatory Commission (NRC) regulations (10 CFR 35.432) require the calibration of newly received brachytherapy sources before the first medical use, there is no regulatory requirement to assay seeds when recycling them. However, it is the institution's responsibility to ensure all seeds are accounted for and returned to the correct lot after each use. In our eye plaque program, during plaque implant and explant, the plaque ID and seed count are visually confirmed by both the medical physicist and the ocular oncologist. Since all seeds on a single plaque are from the same lot, assaying individual seeds is not necessary and would add significant time cost and personnel exposure. Therefore, a batch relative assay, which measures the well chamber response (WCR) of the assembled plaques before implant and after explant, has been implemented to confirm the returned seed batch before placing them into the designated lot.

Prior studies have developed methods to measure the dose rate of a clinical eye plaque in order to verify conformance to the treatment plan.^3^ ^−^ ^5^ However, these methods require specialized equipment with precise setup and are not necessary for relative measurements for plaques with all seeds assembled from the same lot. Currently, as there is no commercially available holder for assaying eye plaques in a well chamber, the reading of a concave plaque placed inside the well chamber holder can be challenging due to variable plaque positioning in the well. 3D printing is already being utilized in medical physics QA procedures to manufacture phantoms.^6^, ^7^ A 3D printed plaque holder placed inside a well chamber could meet the need of batch relative assay.

In this study, we have developed a novel 3D‐printed conical QA holder for batch relative assay and report the results of this new QA method for assay repeatability and reliability. This device permits reproducible placement of the constructed eye plaque for relative response measurements using a well chamber.

## 2 METHODS

### 2.1 3D printing of QA holder

**FIGURE 1:**
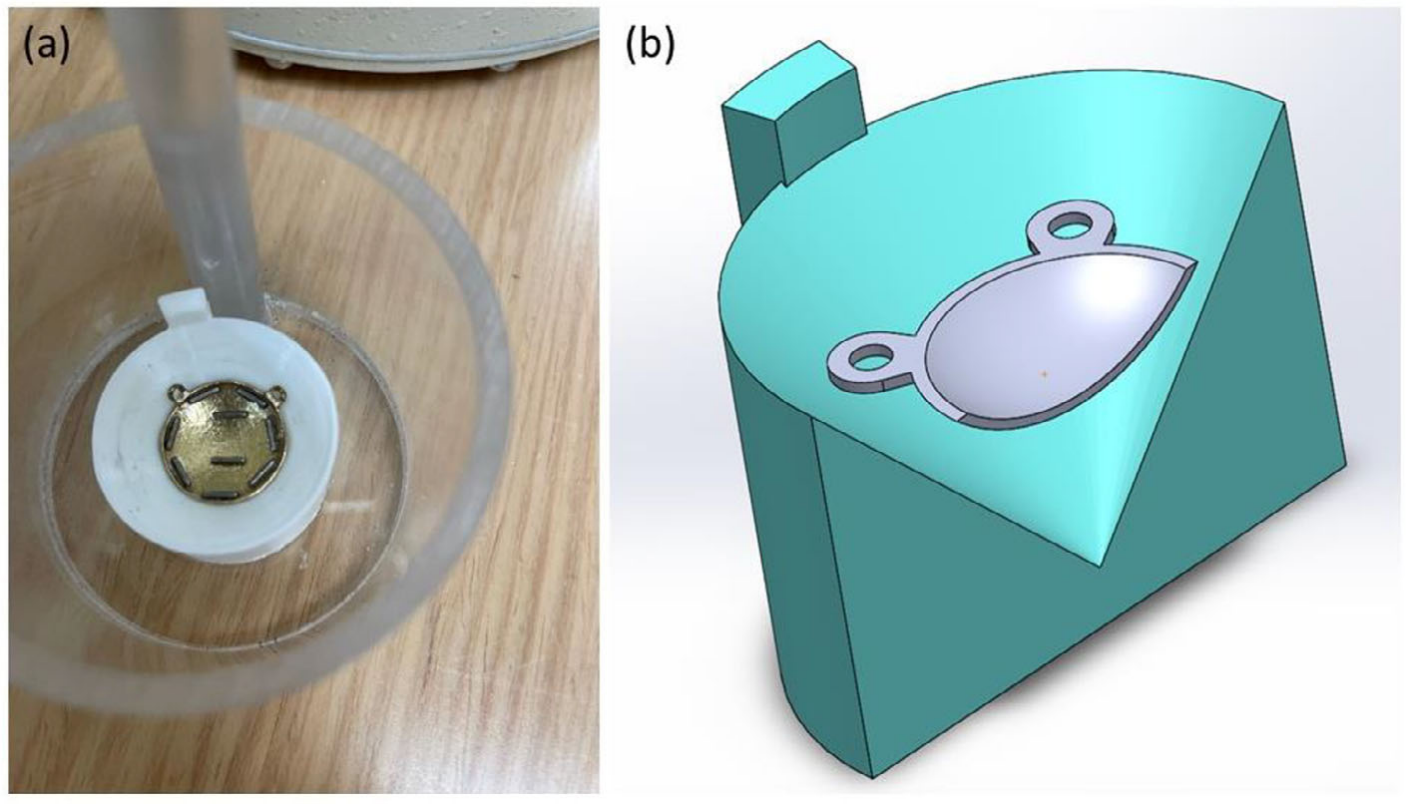
(a) Photograph of an eye plaque in the QA holder placed inside the dipper for the dose calibrator and (b) cross section of the 3D model with eye plaque inside the holder.

SolidWorks 2022 (Dassault Systèmes, Waltham, MA) was used to design a sophisticated 3D model of a universal conical plaque holder. The plaque holder (Figure 1a) has dimensions of 3 cm in diameter and 2 cm in height. This holder accommodates plaques ranging in size from 10 to 22 mm (round, notched, and deep notched), which can be easily placed in a well‐type dose calibrator. A reproducibility test was conducted with test plaques placed in different orientations in the holder as well as without the holder. The conical plaque holder 3D model file was exported as a high resolution STereoLithography (STL) file format and printed using the Ender 3 S1‐Pro 3D printer (Creality, Shenzhen, China). The print material is polylactic acid filament (PLA, 1.75 mm diameter), with 0.4 mm diameter nozzle, 0.2 mm layer height, and 20% infill density. The print temperature was set to a constant 200°C for the nozzle and to 60°C for the build plate, heat settings ideal for PLA.

### 2.2 Eye plaque assembly and measurement

A new lot, each containing 35 seeds with a nominal activity of 5 mCi per seed, is ordered biweekly. The initial new lot assay was performed according to AAPM TG‐221^2^ for 10 of the received seeds. A third‐party source calibration report was also ordered for all 35 seeds. At any one time, there are typically 10 batches of different source strengths available for planning.

Plaque Simulator (Version 6.8.6) treatment planning program (IsoAid, Port Richey, FL) was used for the design and dose evaluation of custom gold plaques using I‐125 seeds (IsoAid, Port Richey, FL). There are a total of 43 unique plaques routinely used in our program, with sizes ranging from 10 to 22 mm and the shapes of round, notched, and deep notched. Each plaque has a unique ID etched on the back and has been scanned into the treatment planning program. As these plaques were handmade by a jeweler, there are subtle geometric differences, such as the plaque thickness, even between plaques with the same size and shape.

I‐125 seeds were loaded directly onto the plaque according to the treatment plan without any seed inserts and affixed with commercially available cyanoacrylate‐containing adhesive. Once the plaque was constructed, a pre‐implant measurement of the plaque with the affixed seeds was made. The plaque was sterilized with benzalkonium chloride solution prior to implantation. The plaque was assayed post explant to confirm that all seeds were accounted for and to confirm the seed lot. A well‐type dose calibrator (Capintec CRC‐15R, Ramsey, NJ) with the printed QA holder was used for plaque assays. The dipper of the dose calibrator (Figure 1b), which was placed in the well for measurements, had a 3‐cm diameter inner circle on its base to accommodate the QA holder securely. It should be noted that although the reading is reported in mCi on the dose calibrator screen, this is a relative WCR of an assembled plaque and not accurate for individual seeds.

### 2.3 Data collection and analysis

From August 2022 to December 2023, a total of 251 eye plaques were assayed with the QA holder before implant and after explant. Plaques were implanted for 4 or 7 days and typically assayed with a 6‐to‐8‐day interval between two measurements. For each plaque, the plaque response fraction (PRF) was calculated with the pre‐implant reading, which is defined as the pre‐implant reading divided by the combined apparent activity of all seeds on the plaque on the same day and can be written as

(1) $$\begin{array}{c} PRF\;=\frac{WCR\left(plaque\right)}{N\left(seed\right)A\left(seed\right)}\;\; \end{array}$$

where WCR(plaque) is the pre‐implant well chamber response of the plaque in millicurie (mCi), N(seed) is the number of seeds on the plaque, and A(seed) is the apparent activity (mCi) of each seed on the day of the measurement. In our eye plaque program, all seeds on one assembled plaque were from the same lot. Over the course of this study, the PRF for each individual plaque is expected to be constant and can be used to confirm the correct seeds have been used by comparing with the average PRF calculated from previous measurements. The second reading was measured after explant, which was compared to the expected value calculated from the pre‐implant reading corrected for decay. A percent deviation was calculated to verify the seeds were returned to the correct lot.

## 3 RESULTS

For the reproducibility test, seven plaques with sizes ranging from 10 to 22 mm were measured at four cardinal angles (0°, 90°, 180°, 270°) relative to the standard position with a repeat measurement for angle 0°. A small degree of tilt was intentionally added when measuring with the plaque holder to simulate suboptimal measurement geometry. In comparison, five additional measurements were made without the QA holder. As can be seen in Table 1, the relative standard deviations were smaller with the use of the QA holder for all plaques except for the 22 mm plaque. The average relative standard deviations for readings with and without the QA holder were 0.40% and 0.68%, respectively. Two‐sample F‐tests were performed to compare the variances between measurements made with and without the conical QA holder. The one‐tailed *p*‐values of the F‐tests were listed in Table 1, which showed statistically significant differences in measurement variances for three of seven plaques.

**TABLE 1:** Well chamber responses (WCR) in the plaque assay reproducibility test.

	Plaque size (mm)	Conical QA holder	WCR 1	WCR 2	WCR 3	WCR 4	WCR 5	Rel. std. dev.	F‐test *p*‐value
Plaque 1	10	Y	9.17	9.14	9.16	9.21	9.17	0.28%	0.164
		N	9.12	9.10	9.13	9.04	9.04	0.48%	
Plaque 2	15	Y	5.09	5.03	5.08	5.07	5.06	0.45%	0.197
		N	5.05	5.01	5.09	5.00	5.02	0.72%	
Plaque 3	18	Y	12.46	12.45	12.45	12.47	12.49	0.13%	0.023^a^
		N	12.49	12.43	12.39	12.35	12.44	0.43%	
Plaque 4	18	Y	9.27	9.26	9.34	9.34	9.29	0.41%	0.079
		N	9.23	9.12	9.30	9.10	9.15	0.91%	
Plaque 5	18	Y	5.18	5.18	5.19	5.20	5.19	0.16%	0.006^a^
		N	5.25	5.23	5.20	5.19	5.15	0.74%	
Plaque 6	20	Y	14.83	14.84	14.83	14.82	14.83	0.05%	<0.001^a^
		N	14.69	14.68	14.62	14.56	14.67	0.37%	
Plaque 7	22	Y	25.5	25.2	25.2	25.8	25.9	1.28%	0.379
		N	25.2	25.3	25.9	25.4	25.3	1.09%	

*Notes*: The reproducibility test compares measurements made with and without the QA holder. The measured plaque sizes ranged from the smallest (10 mm) to the largest (22 mm) available. The well chamber response (WCR) in mCi was measured five times for each setup. The relative standard deviation is calculated as the percentage of the standard deviation of five measurements over the average measurement value. The one‐tailed *p*‐values of the two‐sample F‐tests were calculated for each plaque between measurements made with and without the conical QA holder.

^a^ indicates statistical significance.

**FIGURE 2:**
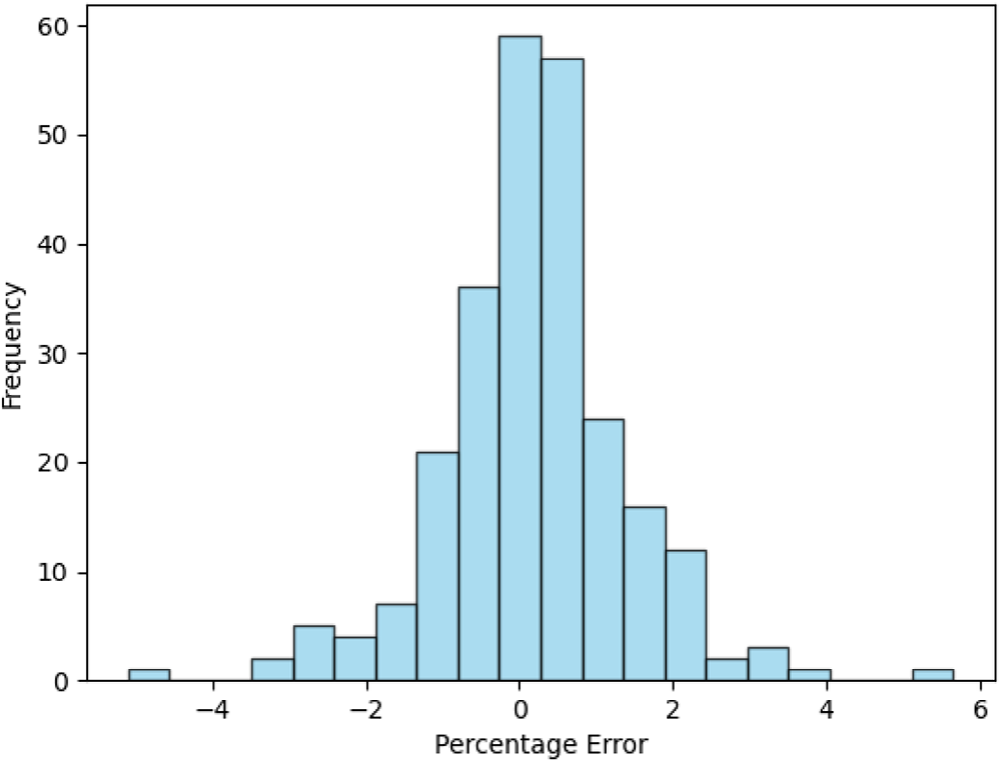
Relative percentage error histogram of explant assay versus expected value. The expected explant assay readings were calculated from the implant assay readings corrected for radioactive decay of I‐125.

For the 251 clinical plaques, the median number of seeds on a plaque was 9, with the range of 4 to 19. The average combined activity of all seeds on the plaque was 34.51 mCi on the day of implant. The relative error between explant assay and expected value was 0.18% ± 1.23% (mean ± standard deviation). Of the 251 clinical plaques, only eight plaques had a relative deviation exceeding ± 3%. The relative error histogram is shown in Figure 2. As the deviation could be positive or negative, the absolute value of measurement deviation was calculated to be 0.89% ± 0.86%.

**FIGURE 3:**
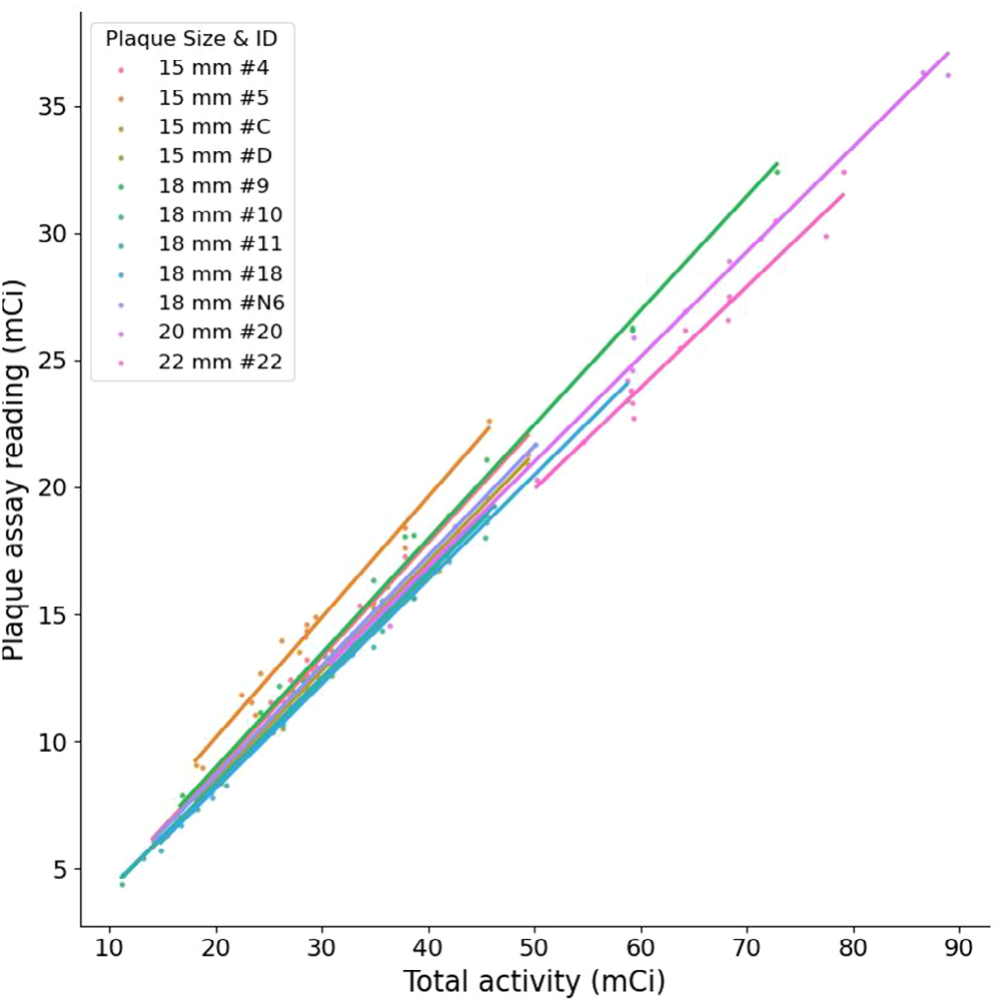
Total seed activity versus plaque assay reading for plaques with at least 10 uses. Each measurement is shown as a point, and a linear fit is displayed for each plaque separately.

The average PRF for all implanted plaques was 43.16% with a standard deviation of 2.98%. For the same plaque, the PRF had less variation, as can be seen from Table 2, which listed the top 3 most used round and notched plaques. Since each plaque had a slightly different amount of shielding provided by the gold alloy backing, the PRFs could be different even for plaques of the same size and shape. For example, although plaques 9 and 10 were both 18 mm round plaques, their average PRFs differed by more than 3 percentage points. Figure 3 shows the plot of total seed activity with the plaque assay reading for plaques with at least 10 uses. Of all the plaques with at least 3 uses, the highest PRF for a single plaque was 49.84% for a 15 mm round plaque with 15 uses, while the lowest PRF was 36.49% for a 22 mm round plaque with 4 uses. The majority of the plaques had a PRF between 40% and 45%. The highest PRF's standard deviation was 2.00% among plaques with at least 3 uses.

**TABLE 2:** Plaque response fraction (PRF) for the three most used round and notched plaques.

Plaque ID	Plaque size and shape	Number of uses	Average PRF	Std. dev.
4	15 mm round	21	44.22%	1.30%
9	18 mm round	20	44.79%	1.90%
10	18 mm round	20	41.46%	1.33%
N6	18 mm notched	12	43.24%	0.72%
N7	18 mm notched	6	42.02%	0.93%
N22	22 mm notched	7	41.88%	0.34%

## 4 DISCUSSION

This study presented the design and implementation of a novel 3D printed QA holder for assaying assembled eye plaques in a well‐type dose calibrator. The advantage of using the QA holder is the ease and consistency of placing the plaque inside the dose calibrator for quick relative batch assay of assembled plaques. With a 3 cm outer diameter, the plaque holder will be able to fit in commonly used well chambers for brachytherapy seed assay, such as the HDR 1000 Plus Well Chamber (Standard Imaging, Middleton, MI) with an insert diameter of 3.5 cm. A dedicated “dipper” would need to be designed to fit in the specific chamber and securely place the plaque holder. Circular plaques (round, notched, or donut shaped) with a cross section up to 23 mm in diameter should fit in the plaque holder. Any plaques larger than that will require a new and larger holder to be printed following the same design concept.

The reproducibility test showed an overall reduced relative standard deviation when the plaque holder was used. It should be noted that the measurements with the plaque holder was made with a random intentional tilt of the plaques, which could introduce a larger uncertainty for larger plaques, such as the 22 mm plaque. To minimize the tilt when placing the plaque, circular circumference marks were drawn on the inner surface of the cone to align different sized plaques. With properly placed plaques, the actual measurement uncertainty is expected to be smaller than that shown in the reproducibility test.

Since the batch relative assay compares the explant reading with the decay corrected pre‐implant reading, possible reasons for measurement uncertainty could include the dose calibrator response, the time of measurement, and plaque placement. The constancy of the dose calibrator can be checked with a standard source and should be within 5% from day‐to‐day. As the two assays of the same plaque could be done at different times of day, there could be as much as 12 h of time difference between the nominal and actual decay time, which amounts to 0.6% difference. It should be noted that appropriate temperature and pressure corrections should be made when using vented well chambers for plaque relative assay. Given the mean and standard deviation of 0.89% and 0.86% of the absolute value of measurement error, we have set the tolerance for batch relative assay at ± 5%. The batch relative assay is able to detect a missing seed between two assays, which is approximately a 5% error for a 20‐seed plaque. Individual seed assays will be performed for plaques exceeding the batch relative assay tolerance.

With sufficient measurement data, the PRF can be used to check if the correct batch of seeds is used in the plaque according to the treatment plan. For a particular plaque, the expected WCR can be calculated from the total seed activity for the current plan and the average PRF from previous measurements. The uncertainty of the PRF includes seed apparent activity and seed distribution on the plaque on top of the measurement uncertainties. According to AAPM TG‐221^2^, the measured activity of the sample mean and individual seed should be within 3% and 6%, respectively. Therefore, we have set the tolerance at ± 10% for plaques with sufficient prior data. Since two consecutive seed lots differ in activity by approximately 14% (2 weeks), an incorrect seed lot can be easily identified during pre‐implant assay. The larger standard deviation of the round plaques compared with the notched plaques could be in part due to different seed distributions. The round plaques may be loaded fully or posteriorly for smaller tumors. The notched and deep notched plaques are typically fully loaded. To use the PRF as a pre‐implant QA requires several measurements to determine the baseline value for each plaque.

This QA procedure cannot replace individual seed assay before recycling of seeds if multiple seed strengths are present on the same plaque. The assay also does not verify the positioning of individual seeds on the plaque. Beiki‐Ardakani et al.^3^ reported a QA system for assembled plaques, which consisted of a pinhole camera with a computed radiography unit to verify the location and relative strength of seeds and a source strength jig with a survey meter to estimate the total seed activity. Weersink et al.^5^ improved on the apparatus by using an optical camera and scintillating film system. The system was able to achieve a 10% accuracy for measuring the plaque's air kerma strength.

## 5 CONCLUSIONS

3D printing is useful to improve QA of eye plaque build process, assuring higher precision of well‐chamber measurements, allowing for efficient verification of seed batch assay. Remarkably, eye plaques with equal numbers of seeds from the same lot number can be distinguished, saving valuable physicist time, reducing radiation exposure from seed handling, while validating all brachytherapy sources were returned to the correct lot.

## AUTHOR CONTRIBUTIONS

The authors confirm contribution to the paper as follows: study conception and design: Firas Mourtada and Jacqueline Emrich; data collection: Jacqueline Emrich and Wentao Wang; analysis and interpretation of results: Wentao Wang, Firas Mourtada, and Jacqueline Emrich; draft manuscript preparation: Wentao Wang, Firas Mourtada, and Jacqueline Emrich. All authors reviewed the results and approved the final version of the manuscript.

## CONFLICT OF INTEREST STATEMENT

The authors declare no conflicts of interest.
